# Relationship Between Human Meibum Lipid Composition and the Severity of Meibomian Gland Dysfunction: A Spectroscopic Analysis

**DOI:** 10.1167/iovs.64.10.22

**Published:** 2023-07-19

**Authors:** Saumya Nagar, Layla Ajouz, Kelly K. Nichols, Sandeep Kumar, Cathy Zhao, Kugen K. Naidoo, Michael R. Robinson, Douglas Borchman

**Affiliations:** 1 Allergan, an AbbVie company, Irvine, CA, United States; 2 School of Optometry, University of Alabama at Birmingham, Birmingham, AL, United States; 3 Department of Ophthalmology and Visual Sciences, University of Louisville, Louisville, KY, United States

**Keywords:** disease severity, dry eye disease, lipid, meibum, meibomian gland dysfunction, cholesteryl ester, wax ester, NMR

## Abstract

**Purpose:**

Information on the relationship between meibum lipid composition and severity of meibomian gland dysfunction (MGD) is limited. The purpose of this study was to analyze the molecular components of meibum collected from individuals with no MGD, mild-to-moderate MGD, and severe MGD.

**Methods:**

Adults with and without MGD were enrolled in a prospective, multicenter, exploratory clinical trial (ClinicalTrials.gov Identifier: NCT01979887). Molar ratios of cholesteryl ester to wax ester (R_CE/WE_) and aldehyde to wax ester (R_ald/WE_) in meibum samples were measured with ^1^H-NMR spectroscopy. Results were evaluated for participants grouped by MGD disease status and severity (non-MGD, mild-to-moderate MGD, and severe MGD), as defined by maximum meibum quality scores, Schirmer test results, and Subject Ocular Symptom Questionnaire responses.

**Results:**

Sixty-nine meibum samples from 69 individuals were included in the analysis: 24 non-MGD, 24 mild-to-moderate MGD, and 21 severe MGD. Mean R_CE/WE_ was 0.29 in non-MGD, 0.14 in mild-to-moderate MGD (*P* = 0.038 vs. non-MGD, 51% lower), and 0.07 in severe MGD (*P* = 0.16 vs. mild-to-moderate MGD, 52% lower; *P* = 0.002 vs. non-MGD, 76% lower). Mean R_ald/WE_ was 0.00022 in non-MGD, 0.00083 in mild-to-moderate MGD (*P* = 0.07 vs. non-MGD, 277% higher), and 0.0024 in severe MGD (*P* = 0.003 vs. mild-to-moderate MGD, 190% higher; *P* < 0.001 vs. non-MGD, 992% higher).

**Conclusions:**

R_CE/WE_ was lowest and R_ald/WE_ was highest in the severe MGD cohort, suggesting that these meibum constituent molar ratios may result from the pathophysiology associated with MGD and can impact ocular surface lipid and tear film homeostasis. These findings may potentially help identify targets for MGD treatment.

Dry eye disease (DED) is a common ocular condition that has been defined by the Tear Film and Ocular Surface Society Dry Eye Workshop II (TFOS DEWS II) as “a multifactorial disease of the ocular surface, characterized by loss of homeostasis of the tear film and accompanied by ocular symptoms, in which an etiological role is played by instability and hyperosmolarity of the tear film, inflammation and damage to the ocular surface, and neurosensory abnormalities.”^1^ The dominant mechanism of DED is aqueous evaporation from the tear film leading to a hyperosmolar tear film and subsequent tissue damage.^2^ Multiple treatment options are currently available including topical lubricants, anti-inflammatory medications (e.g., cyclosporine, corticosteroids, lifitegrast), oral antibiotics, at-home and in-office heat therapy, intense-pulsed light therapy, and punctal occlusion.^3^

The tear film lipid layer (TFLL) is a thin, viscoelastic film of lipid that is located at the superficial surface of the tear film and has many important functions related to DED.^4^^,^^5^ It is thought that the structural organization of the TFLL consists of nonpolar lipids at the air-tear interface and amphiphilic polar lipids adjacent to the aqueous-mucin layer of the tear film.^6^ The TFLL is not homogeneous, but instead, varies in thickness across the ocular surface.^7^ Ellipsometry measurements using tear samples in vitro have suggested that the TFLL thickness may be 0 to 2.6 nm in thin areas and ∼200 to 500 nm in thick areas.^8^ The TFLL aids in the spreading of tears and ocular surface lubrication; upon blinking, the TFLL is drawn upward and the tear film spreads, driven by the Marangoni effect.^9^^,^^10^ The TFLL also serves as a barrier to evaporation and overspill of tears. Furthermore, it is needed to reduce surface tension and maintain tear film stability.^4^^,^^5^^,^^9^^,^^11^^–^^13^ Importantly, changes in the composition and structure of the TFLL are believed to contribute to the instability of tears in DED.^10^^,^^14^^–^^16^

The TFLL is largely composed of meibomian gland secretions (meibum).^5^ The meibomian glands are modified sebaceous glands in the eyelids^17^ that secrete meibum consisting of a complex mixture of lipids: *>*90% are nonpolar lipids including wax esters (WE), sterol esters (mainly cholesteryl esters [CE]), free cholesterol, triacylglycerols, diesters, and squalene; and *<*10% are polar lipids including (O-acyl)-ω-hydroxy fatty acids, free fatty acids, and phospholipids.^17^^–^^26^ Meibomian gland dysfunction (MGD), a disorder of the meibomian glands characterized by terminal duct obstruction or by alterations in the quality or quantity of secreted meibum, is a leading cause of DED.^17^^,^^27^

Nuclear magnetic resonance (NMR) spectroscopy has been widely used to analyze the lipid composition in human meibum and is a valuable tool for evaluating the relationships between tear film lipid composition, structure, and function.^10^ Principal component analysis of infrared and NMR spectra of meibum has shown both age-related and MGD-related changes in meibum composition that could result in reduced meibum quality and decreased function of the TFLL.^28^^–^^30^ Notably, CE and WE are the main lipid species in meibum.^18^^–^^23^ The hydrocarbon chains of CE in meibum are among the longest measured for lipids, up to 32 or 34 carbons in length.^18^^,^^31^^,^^32^ CE contain more anteiso- and iso-branched hydrocarbon chains compared with WE, and the hydrocarbon chains of CE are more saturated compared with WE.^18^^,^^33^ The molar ratio of CE to WE (R_CE/WE_) in meibum influences structural changes and the rheology of the surface film formed by meibum lipid (i.e., the maximum surface pressure attained at minimal surface area) and the transient dilatational modulus.^34^ Importantly, NMR and infrared spectroscopic studies have shown that R_CE/WE_ in meibum is approximately 0.49 in human control subjects but is decreased in patients with MGD.^35^^–^^37^

There is limited information on the relationship between the molecular composition of meibum and MGD disease severity. The purpose of this study was to analyze by NMR and compare the molecular composition of meibum collected from individuals with no MGD, mild-to-moderate MGD, and severe MGD.

## Materials and Methods

### Clinical Study Design

This analysis used meibum samples obtained in a three-week, prospective, multicenter (three clinical sites), observational, exploratory study in individuals with and without MGD (ClinicalTrials.gov Identifier: NCT01979887). The study was conducted in accordance with the Declaration of Helsinki and applicable regulations. At each study site, an institutional review board or ethics committee approved the study protocol before the study was initiated, and all participants provided written informed consent before screening.

**Figure 1.**
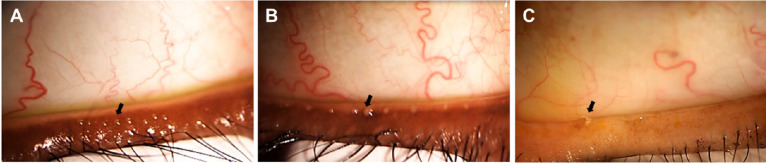
Photographs of expressed meibum (arrows) in representative study participants with and without MGD. (**A**) Meibum has an olive oil–type consistency/viscosity and is expressed easily in participants without MGD. In participants with mild-to-moderate MGD (**B**) and severe MGD (**C**), meibum exhibits a turbid or toothpaste-type consistency.

Individuals potentially eligible for the study were enrolled, and those who qualified to participate in the study by meeting cohort entry criteria were allocated into the 3 study cohorts: non-MGD, mild-to-moderate MGD, and severe MGD, in accordance with diagnostic criteria and severity grading described in the executive summary from the International Workshop on Meibomian Gland Dysfunction.^38^ Allocation of individuals to cohorts was planned to continue until approximately 25 individuals were assigned to each cohort. The cohort selection criteria included maximum meibum quality scores, Schirmer test results, and Subject Ocular Symptom Questionnaire responses (Table 1). Meibum quality was normal in the non-MGD cohort, poorer in the mild-to-moderate MGD cohort, and worst in the severe MGD cohort (Fig. 1). Details of clinical diagnosis and clinical outcome measures in the cohorts will be reported elsewhere (manuscript in preparation).

**Table 1.** Selection Criteria for Cohorts at the Enrollment Study Visit

Cohort	MMQS^*^^,^^†^	Schirmer Test Without Anesthesia^†^	Sum of Worst 2 Symptom Scores on the Subject Ocular Symptom Questionnaire^‡^
Non-MGD	0 or 1^§^	≥7 mm/5 min	0 to 4 with neither symptom scored as >2
Mild-to-moderate MGD	2^§^	≥7 mm/5 min	0 to 4 with neither symptom scored as >2
Severe MGD	3	≥7 mm/5 min	≥4

MGD, meibomian gland dysfunction; MMQS, maximum meibum quality score.

Selection criteria from the latest protocol amendment are listed. The MMQS was graded by the investigator; higher scores indicate lower quality of the secretion.

* Only six central glands in the lower lids were examined and graded.

† MMQS and Schirmer test criteria must be met in the same eye (the study eye providing the meibum sample analyzed).

‡ Scored by the study participant.

§ No gland among the six central glands being graded may have a meibum quality score greater than the MMQS specified in the cohort designation.

### Collection of Meibum Samples and NMR Analysis

Meibum samples were collected from study participants at the study exit visit. After meibum was expressed by application of uniform pressure on the lower eyelid using the Meibomian Gland Evaluator (Johnson & Johnson Vision, Irvine, CA, USA) and the meibomian gland secretion quality was graded, the investigator collected meibum from the 6 central glands of the lower lid of each eye using Sebutape (Clinical and Derm, Dallas, TX, USA) and a meibum collection kit (Supplementary Fig. S1), as described in Supplemental Methods. Separate samples were collected for the right and left eyes. The samples from the study eyes were shipped on dry ice and stored at −20°C for NMR analysis.

For NMR analysis, the Sebutape was removed from the meibum collection kit and placed into a 9-mm prelabeled microvial with a Teflon cap (Microliter Analytical Supplies, Suwanee, GA, USA). The vial was filled with argon gas (10 seconds, gentle flow adjusted by a needle valve, 100 KPa, Analyzed, Ultra-Pure; Welders Supply, Louisville, KY, USA) and 0.5 mL deuterated chloroform (Sigma-Aldrich, St. Louis, MO, USA) and was then sonicated in an ultrasonic bath (Branson Ultrasonics, Sterling Heights, MI, USA) for 10 minutes. The solution was transferred within a few hours to a prelabeled glass NMR tube (Sigma-Aldrich) for immediate NMR analysis.

A maximum of 10 samples were analyzed at a time. Accompanying every set of samples run in the NMR, a standard of deuterated chloroform containing 5 µL tetramethylsilane (Sigma-Aldrich) per mL deuterated chloroform was run to lock and calibrate the instrument. Spectra were acquired with a minimum of 1,250 scans, 45° pulse width, and a relaxation delay of 1.000 second over a period of approximately 1.5 hours, providing sufficient time to collect enough spectra to maximize the signal-to-noise ratio. All spectra were obtained at 25°C. Each raw data file per sample was saved using a unique identifier name. After analysis of the samples was completed, the sample was loaded back into the original 9-mm microvial with Teflon cap that had been used prior to the sample being transferred into the NMR tube.

Spectral data were acquired using a Varian VNMRS 700 MHz NMR spectrometer (Varian, Lexington, MA) equipped with a 5 mm ^1^H[^13^C/^15^N] ^13^C enhanced PFG cold probe (Palo Alto, CA). Tetramethylsilane was used as a 0 ppm reference. The CDCl_3_ resonance at 7.24 ppm was used to confirm the shift value of the samples and standard. Commercial software (GRAMS 386; Galactic Industries Corp., Salem, NH, USA) was used for phasing, curve fitting, and integrating.

### Measurement of Molar Ratios of CE and Aldehyde Relative to WE

R_CE/WE_ was measured as previously described^35^ using the following formulas where I is the integrated intensity of the resonance:

(1) $$\begin{array}{c} R_{\mathrm{CE}/\mathrm{WE}}=\left(I_{4.6}/I_{4.0}\right)\times 2 \end{array}$$

(2) $$\begin{array}{c} R_{\mathrm{CE}/\mathrm{WE}}=\left[\left(I_{1.0}+I_{0.63}\right)/I_{4.0}\right]/3 \end{array}$$

Equation 1 was used for the initial analysis; post hoc analyses were performed using each equation. The molar ratio of aldehyde to wax ester (R_ald/WE_) was measured with the formula R_ald/WE_ = I_9.8_/I_4.0_ where I is the integrated intensity of the resonance.^39^

### Statistical Analysis

In this exploratory analysis, data are shown as mean ± standard error of the mean. *P* values based on Student *t* tests are provided for reference without adjustment for multiplicity.

## Results

Seventy-five adults who were enrolled in the study met the criteria for cohort selection and were classified into the non-MGD (n = 25), mild-to-moderate MGD (n = 25), and severe MGD (n = 25) cohorts. Among these 75 participants, the mean (standard deviation) age was 54.5 (9.49) years, and 66.7% (50/75) were female. Most of the 75 participants were Black (44.0%, 33/75) or White (30.7%, 23/75).

### NMR Analysis of Meibum Composition

**Figure 2.**
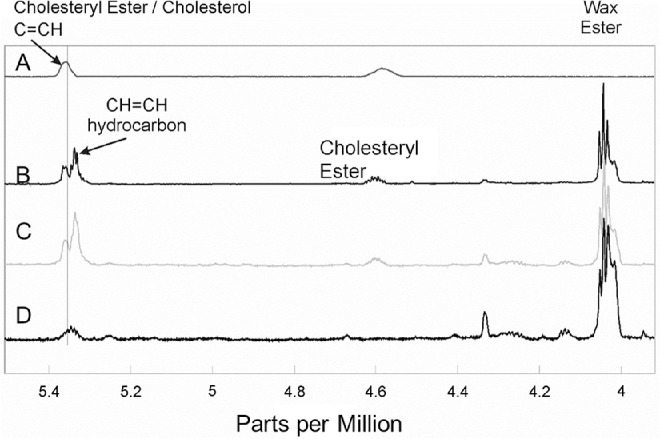
^1^H-NMR spectra. (**A**) Typical spectrum of cholesteryl stearate; (**B**) spectrum of meibum expressed and pooled from four lids of an eight-year-old child without ocular disease; and (**C**, **D**) average spectra of meibum for participants in this study whose spectra (**C**) contained or (**D**) did not contain measurable cholesteryl ester in the initial analysis. The y-axis unit is resonance intensity, and the spectra were scaled and shifted along the y axis individually.

NMR evaluation of the meibum samples from the study eyes was performed in a laboratory that was masked to the cohort assignment of each sample. NMR spectra were obtained for samples from 73 of the 75 study eyes (one sample was not properly shipped to the analysis facility, and for one other sample, there were technical difficulties locking onto the NMR signal). Spectra for a total of 69 meibum samples were analyzable and included in the analysis: 24 each from the non-MGD and mild-to-moderate MGD cohorts and 21 from the severe MGD cohort. The NMR spectra were typical of published spectra of human meibum (Fig. 2B). Five major resonances were observed in the ester region: the C = CH resonance at 5.36 ppm associated with carbon 6 on cholesterol and cholesterol-related molecules, the resonance at 5.34 ppm from hydrocarbon = CH moieties, the CE resonance at 4.6 ppm, the glyceryl ester resonance near 4.14 ppm, and the WE resonance near 4.0 ppm. Spectra could be grouped into samples containing a measurable CE resonance (Fig. 2C) and those without a measurable CE resonance (Fig. 2D). The mean quantity of esters per meibum sample ranged from 49 to 79 nmoles across the three cohorts (Table 2).

**Table 2.** Relative and Total Quantities of Lipid Moieties in Human Meibum

Cohort	Molar Ratio of CE/WE (Samples With Measurable CE)	Molar Ratio of CE/WE (All Samples)	Total Esters Per Sample (nmol)
Non-MGD	0.46 ± 0.06 (15)	0.29 ± 0.06 (24)	49 ± 6
Mild-to-moderate MGD	0.31 ± 0.06 (11)	0.14 ± 0.04 (24)	79 ± 14
Severe MGD	0.17 ± 0.04 (9)	0.07 ± 0.02 (21)	62 ± 11
*P*, non-MGD vs mild-to-moderate MGD	0.077	0.038	
*P*, mild-to-moderate vs severe MGD	0.062	0.16	
*P*, non-MGD vs severe MGD	0.0015	0.0016	

CE, cholesteryl ester; WE, wax ester.

Data are mean ± standard error of the mean. The number of samples is shown in parentheses.

**Figure 3.**
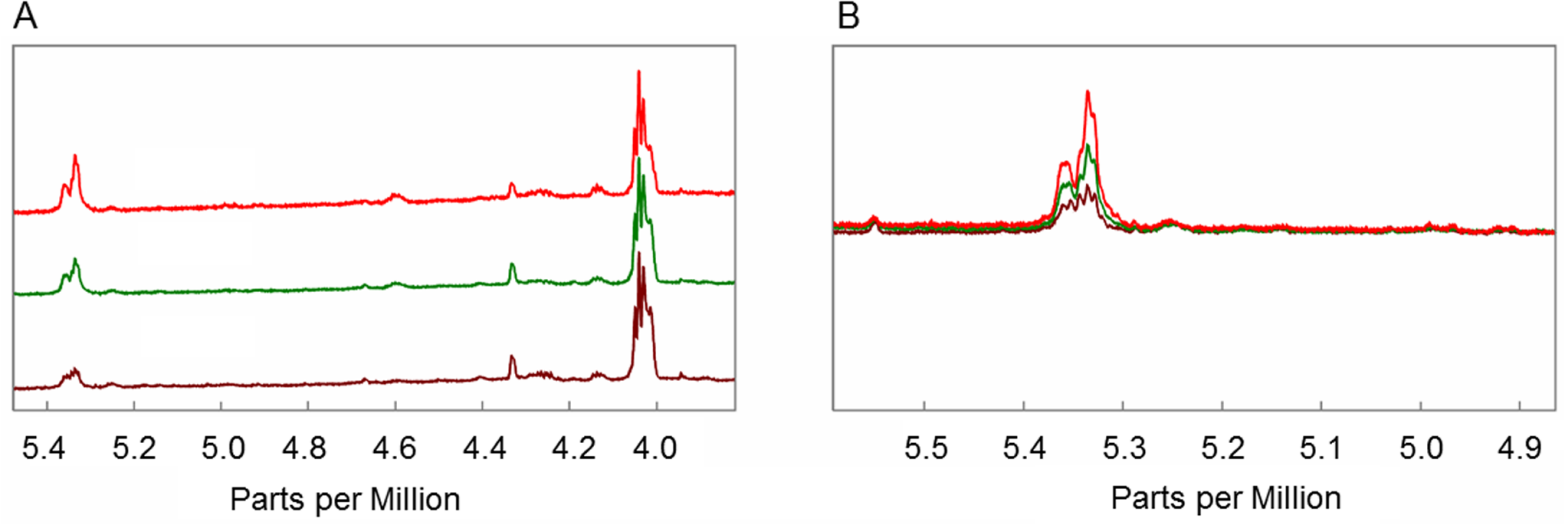
Average ^1^H NMR spectra of human meibum. Resonance assignments are: unsaturated hydrocarbon chains at 5.34 ppm, C = CH cholesterol/cholesteryl ester resonance at 5.36 ppm, cholesteryl ester resonance at 4.6 ppm, glyceryl ester resonance near 4.14 ppm, and wax ester resonance near 4.0 ppm. (**A**) Ester region of the ^1^H NMR spectra. (**B**) Cholesteryl/cholesterol ester and unsaturated hydrocarbon chain resonance intensities relative to the wax ester resonance intensity. *Red,* non-MGD; *green*, mild-moderate MGD; *brown*, severe MGD. The y-axis unit is resonance intensity, and the spectra were scaled and shifted along the y axis individually.

**Figure 4.**
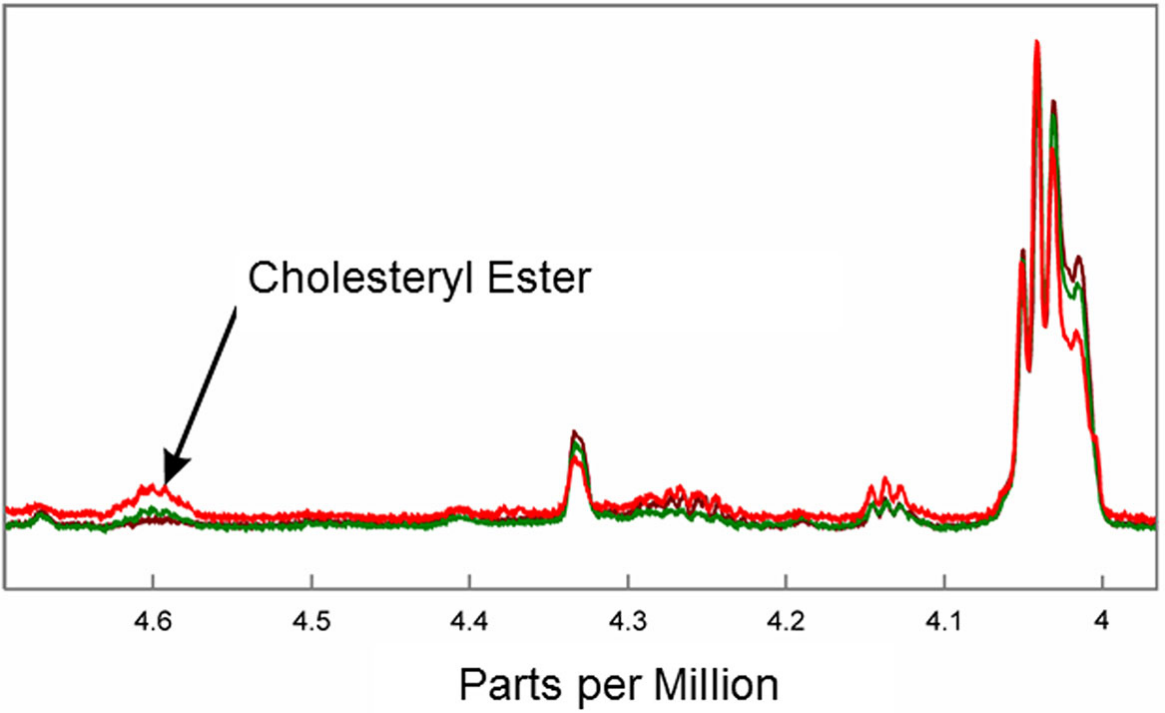
Average ^1^H NMR spectra of human meibum. Resonance assignments are: cholesteryl ester resonance at 4.6 ppm, glyceryl ester resonance near 4.14 ppm, and wax ester resonance near 4.0 ppm. The y-axis unit is resonance intensity, and the spectra were scaled and shifted along the y axis individually. *Red,* non-MGD; *green*, mild-moderate MGD; *brown*, severe MGD.

**Figure 5.**
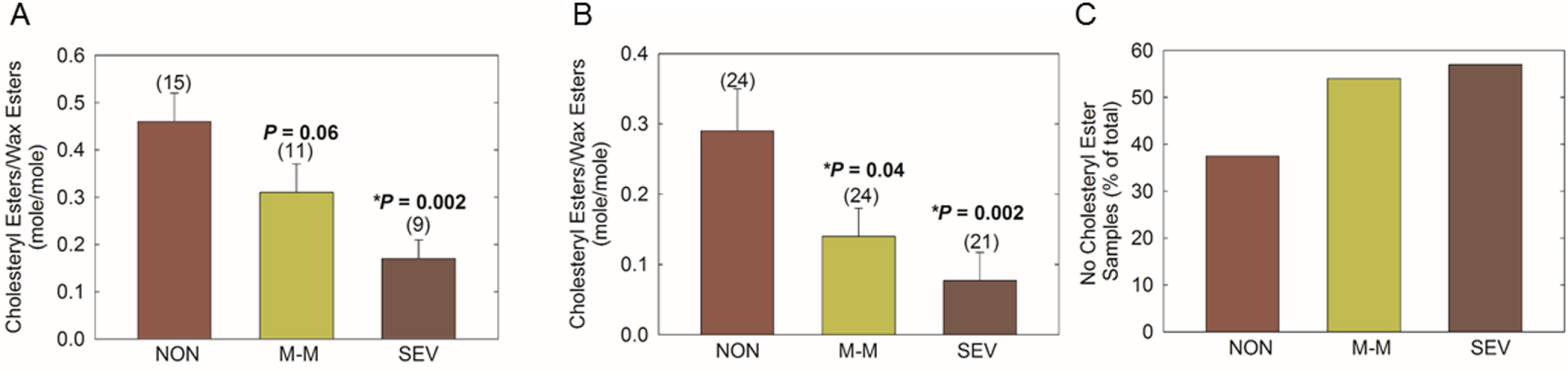
Molar ratio of cholesteryl ester to wax ester in meibum samples from the non-MGD (NON), mild-to-moderate MGD (M-M) and severe MGD (SEV) cohorts. (**A**) Mean molar ratio of cholesteryl ester to wax ester with all samples included. (**B**) Mean molar ratio of cholesteryl ester to wax ester with only samples that had measurable cholesteryl ester included. (**C**) Percentage of samples that had no measurable cholesteryl ester. *P* values shown are vs non-MGD. Values in parentheses are the number of samples. *Error bars:* standard error of the mean. CE, cholesteryl ester.

There were significant differences in the average ^1^H NMR spectra in the ester region among the three cohorts (Fig. 3). The intensity of the cholesterol/CE and hydrocarbon chain HC = CH resonances near 5.35 ppm relative to the intensity of the WE resonance at 4.01 ppm was higher in the NMR spectra of meibum from non-MGD participants than in the spectra of meibum from participants with mild-to-moderate and severe MGD (Fig. 3B). The intensity of the CE resonance at 4.6 ppm was higher in non-MGD samples compared with mild-to-moderate MGD samples and higher in mild-to-moderate MGD samples compared with severe MGD samples (Fig. 4). Mean R_CE/WE_ followed the trend non-MGD > mild-to-moderate MGD > severe MGD (Fig. 5A, Table 2). Mean R_CE/WE_ was 51% lower (*P* = 0.038) for mild-to-moderate MGD compared with non-MGD, 52% lower (*P* = 0.16) for severe MGD compared with mild-to-moderate MGD, and 76% lower (*P* = 0.002) for severe MGD compared with non-MGD. Some of the difference in R_CE/WE_ observed between the non-MGD and mild-to-moderate MGD cohorts (Fig. 5A) was due to a greater percentage of samples with no CE in mild-to-moderate MGD (Fig. 5C). However, mean R_CE/WE_ followed the trend of non-MGD > mild-to-moderate MGD > severe MGD even when only samples with measurable CE were included in the analysis (Fig. 5B, Table 2).

**Figure 6.**
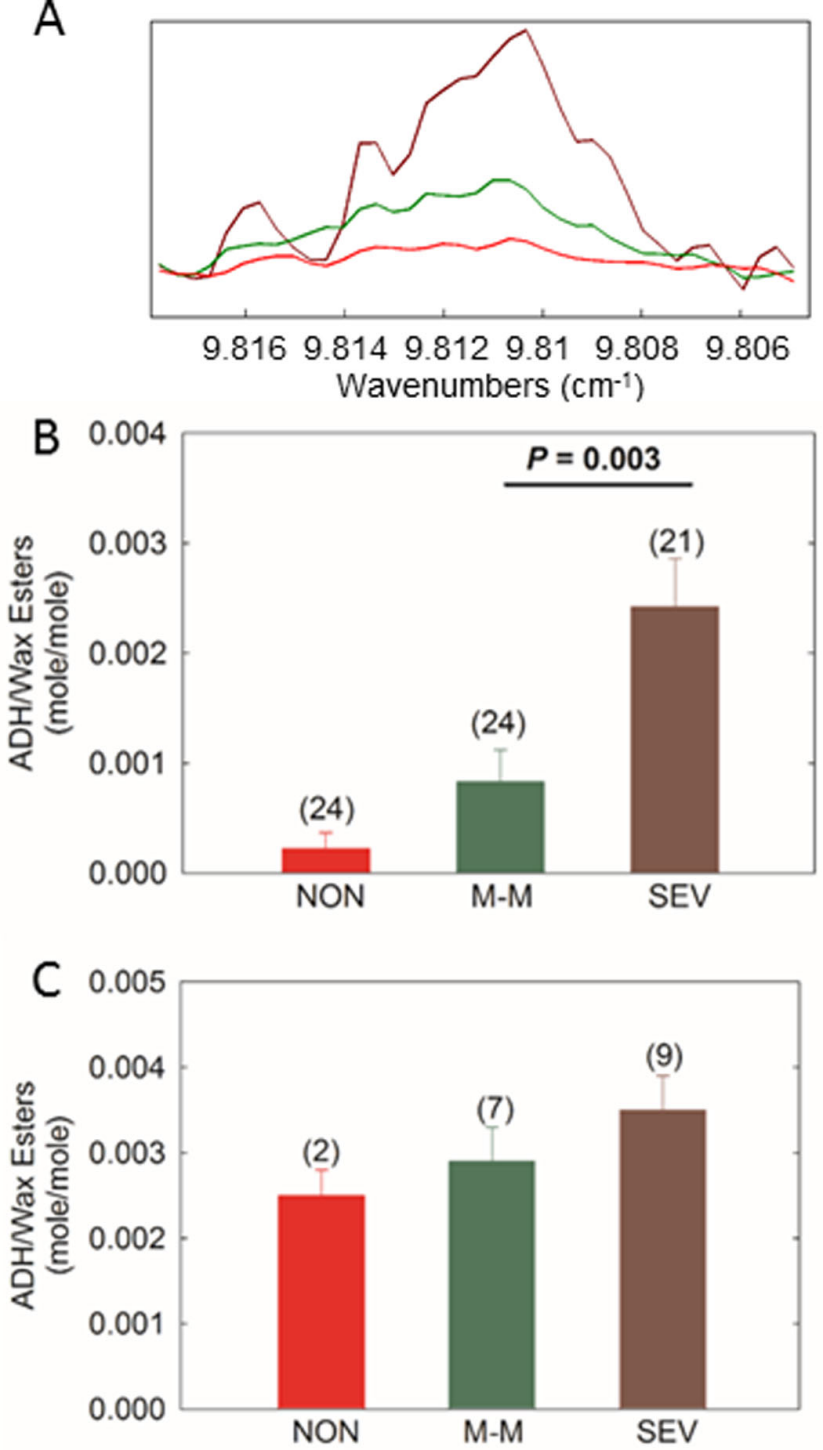
Analysis of aldehydes in meibum. (**A**) Average ^1^H NMR spectra of meibum in the non-MGD (NON, *red*), mild-to-moderate MGD (M-M, *green*) and severe MGD (SEV, *brown*) cohorts. The y-axis unit is resonance intensity, and the spectra were scaled and shifted along the y axis individually. (**B**) Mean molar ratio of aldehyde to wax ester with all samples included. (**C**) Mean molar ratio of aldehydes to wax ester with only samples that had measurable cholesteryl ester included. Values in parenthesis are the number of samples. *Error bars:* standard error of the mean. ADH, aldehyde.

Aldehydes (near 9.8 ppm) (Fig. 6A) were detected in two, seven, and nine samples in the non-MGD, mild-to-moderate MGD, and severe MGD cohorts, respectively. The average signal for aldehydes in the meibum samples followed the trend severe MGD > mild-to-moderate MGD > non-MGD. Mean R_ald/WE_ was 277% higher (*P* = 0.07) for mild-to-moderate MGD compared with non-MGD, 190% higher (*P* = 0.003) for severe MGD compared with mild-to-moderate MGD, and 992% higher (*P* < 0.001) for severe MGD compared with non-MGD (Fig. 6B). When averaging only the samples with detectable aldehydes, mean R_ald/WE_ followed the trend severe MGD > mild-to-moderate MGD > non-MGD (Fig. 6C).

### Post Hoc Analysis of R_CE/WE_ Using the NMR Spectra

**Figure 7.**
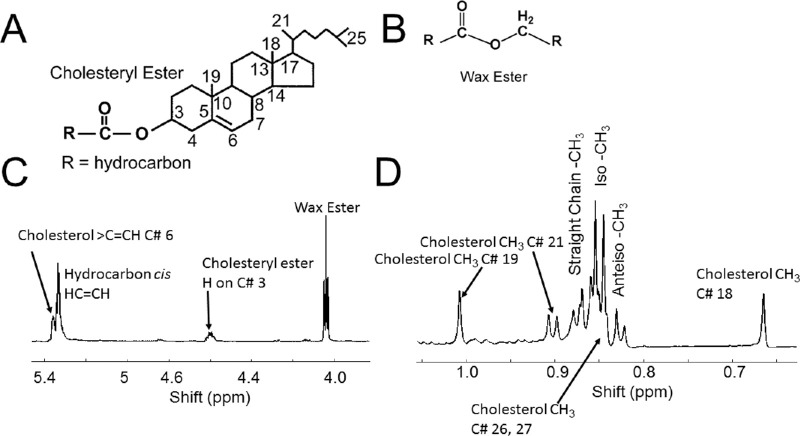
(**A**) Structure of cholesteryl ester. (**B**) Structure of wax ester. The protons shown exhibit a resonance near 4.0 ppm in **C**. (**C**, **D**) A typical NMR spectrum of meibum from a 31-year-old male White donor. The numbering is associated with cholesteryl ester carbons numbered in **A**. The resonance associated with cholesterol number C21 is a doublet.

The analysis above was performed at the time of study completion in 2014. Since then, a number of advances in measuring R_CE/WE_ using ^1^H-NMR spectroscopy prompted post hoc analysis of the spectra (see Discussion for details). For instance, the resonances near 1 and 0.63 ppm are due to protons on carbons 18 and 19 in cholesterol and cholesterol-related molecules (Fig. 7). They are six times more intense than the single proton resonance at 4.6 ppm used in the initial analysis. The post hoc analysis using the resonances at 1.0 and 0.63 ppm showed that R_CE/WE_ was significantly lower (*P* < 0.0001) in samples from participants with MGD compared with samples from participants without MGD (Fig. 7, Table 3). The results were consistent with those of the initial analysis (Table 2), with mean R_CE/WE_ following the trend non-MGD > mild-to-moderate MGD > severe MGD in each post hoc analysis.

**Table 3.** Post Hoc Analyses of the Cholesteryl Ester/Wax Ester Molar Ratio in Human Meibum

Cohort	Molar Ratio of CE/WE^*^ (Samples With Measurable CE)	Molar Ratio of CE/WE^*^ (All Samples)	Molar Ratio of CE/WE^†^
Non-MGD	0.51 ± 0.03 (13)	0.28 ± 0.06 (22)	0.38 ± 0.05 (22)
Mild-to-moderate MGD	0.36 ± 0.05 (8)	0.14 ± 0.04 (21)	0.25 ± 0.03 (21)
Severe MGD	0.23 ± 0.04 (5)	0.06 ± 0.03 (21)	0.21 ± 0.03 (21)
All MGD (mild-to-moderate + severe)	0.31 ± 0.04 (13)	0.10 ± 0.03 (42)	0.23 ± 0.02 (42)
*P*, non-MGD vs mild-to-moderate MGD	0.013	0.06	0.44
*P*, mild-to-moderate vs severe MGD	0.096	0.12	0.39
*P*, non-MGD vs severe MGD	<0.0001	0.002	0.0007
*P,* non-MGD vs all MGD	0.0005	0.0038	<0.0001

CE, cholesteryl ester; WE, wax ester.

The post hoc analyses used a curve-fitting algorithm to more accurately integrate the 4.0 ppm triplet peak. Data are mean ± standard error of the mean. The number of samples is shown in parentheses.

* Calculated using the 4.6 and 4.0 ppm resonances, as in the initial analysis.

† Calculated using the 1.0, 0.63 and 4.0 ppm resonances; all samples had measurable CE.

The spectra of all samples, even those without a measurable CE resonance at 4.6 ppm, had peaks at 1 and 0.63 ppm. Data from post hoc analysis using samples with and without the 4.6 resonance are shown in Table 5. The R_CE/WE_ in samples with no detectable 4.6 ppm resonance was significantly lower, *P* < 0.01, compared with samples with a detectable 4.6 ppm resonance. For the current study, given the paucity of sample from only six meibomian glands, the 4.6 ppm resonance was not a good resonance for calculating R_CE/WE_ when the ratio was less than about 0.24. The 4.6 ppm resonance was useful in previous studies when all meibomian glands of the individual were expressed.^30^^,^^39^

## Discussion

There is sparse information in the literature on the relationship between meibum lipid composition and the severity of MGD. This study showed for the first time that a link exits between R_CE/WE_ in meibum and MGD disease severity through masked analysis of data from a large study population. It has been reported previously that the R_CE/WE_ is reduced in the meibum of patients with MGD.^35^^–^^37^^,^^40^ This study confirms and extends the previous findings by showing a progressively lower R_CE/WE_ ratio in meibum with increasing MGD disease severity. Furthermore, R_ald/WE_ in meibum was identified as a potential marker of MGD disease severity.

To our knowledge, a relative increase in aldehyde in the meibum of individuals with MGD has not been reported previously. Aldehydes including reactive aldehyde species such as malondialdehyde can result from lipid peroxidation. Reactive aldehyde species are proinflammatory^41^ and are believed to be involved in the pathophysiology of DED, as levels of malondialdehyde in the tears of patients with DED have been shown to correlate with both signs and symptoms of dry eye.^42^ An aldehyde trap currently is in development for treatment of DED.^43^ Our findings of increased aldehyde levels in the meibum of individuals with MGD suggest the possibility that aldehydes may distribute from the meibum into the aqueous tear film and have inflammatory effects in DED associated with MGD.

**Figure 8.**
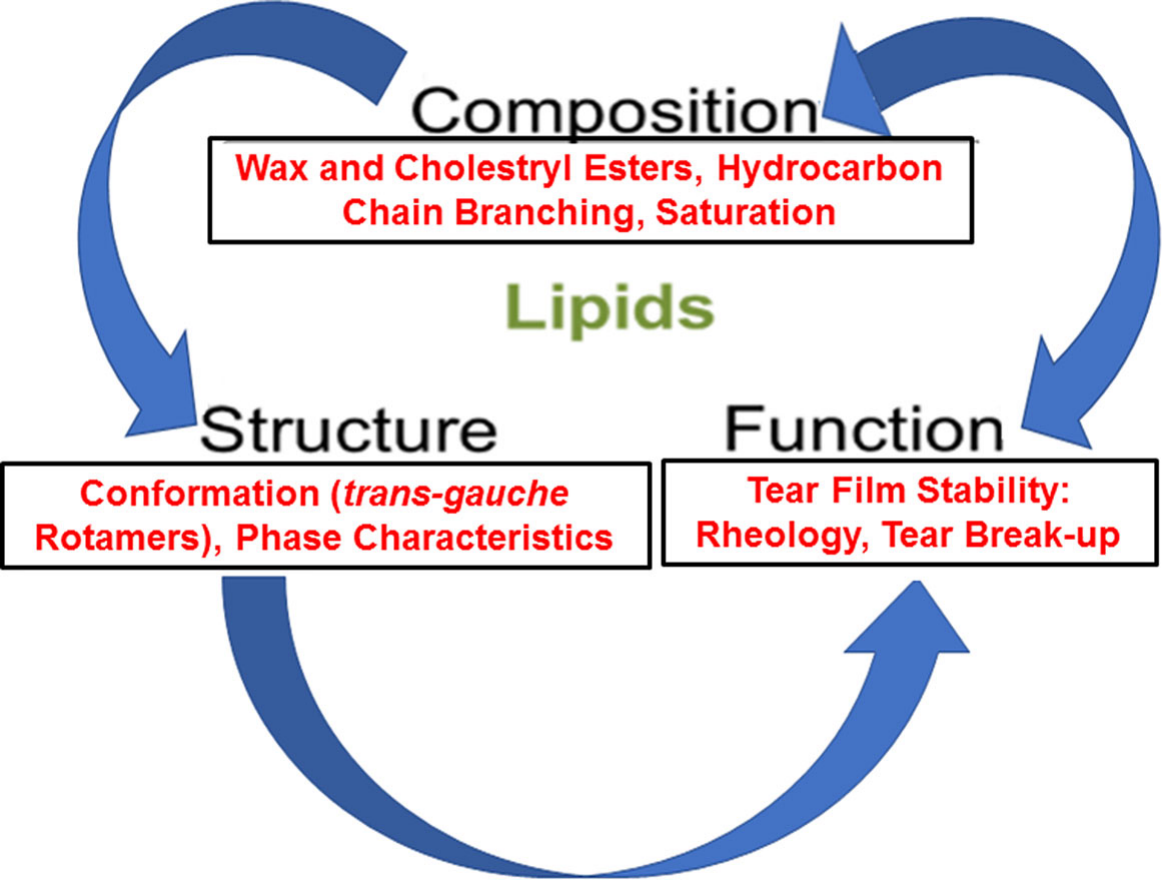
Our hypothesis is that meibum compositional differences contribute to meibum structural differences, which contribute to meibum functional differences observed with age and dry eye.

Analysis of the composition of meibum is important for understanding how the quality of meibum influences tear film stability (Fig. 8). Studies using human meibum have suggested that CE-WE interactions in the meibum have an important role in the structure and function of the TFLL.^10^^,^^37^^,^^44^ In addition, multiple studies using animal models targeting key genes and enzymes involved in the biosynthesis of meibum have shown that reduced CE levels are associated with MGD-like symptoms.^45^^–^^47^

### Cause and Consequences of a Lower R_CE/WE_ in Meibum

The cause of the decrease in meibum R_CE/WE_ in MGD is unknown. It is not due to hydrolysis of CE because the R_CE/WE_ calculated using the CE resonance at 4.6 ppm (which is due exclusively to CE) was always larger compared with R_CE/WE_ calculated using the resonances at 1 ppm and 0.63 ppm (which are due to cholesterol/CE). Had hydrolysis of CE occurred, R_CE/WE_ calculated using the CE resonance at 4.6 ppm would have been smaller, not larger, compared with R_CE/WE_ calculated using the resonances at 1 ppm and 0.63 ppm. It is possible that the decrease in R_CE/WE_ is due to the inflammation that occurs in DED,^1^ because inflammation can result in decreased cholesterol synthesis.^48^

Our previous research has shown a relationship between meibum structure (conformation) and function: more ordered (stiff) meibum is associated with a decrease in tear film stability.^10^ A major question is, what compositional changes cause meibum order to increase in DED? R_CE/WE_ is a major compositional variant in meibum, and we have found that meibum R_CE/WE_ levels are associated with dry eye in patients with Parkinson's disease,^49^ MGD,^35^^–^^37^ and Sjögren's syndrome,^50^ as well as in patients who received local plaque brachytherapy for choroidal melanoma.^51^ Furthermore, changes in R_CE/WE_ were shown to influence the rheology of tear lipids on an aqueous surface in vitro,^34^ increasing the probability that it contributes to, rather than is a consequence of, evaporative DED. However, the answer to the question of cause or effect is not as simple as “a loss of CE increases lipid order, resulting in a loss of tear film stability and DED.” Meibum R_CE/WE_ levels are elevated, rather than decreased, in patients with Sjögren's syndrome and dry eye.^50^ Furthermore, when CE and WE were collected from human meibum and mixed together in different ratios, it was found that decreases in CE can either order or disorder the lipid mixture, depending on whether the WE was more or less ordered than the CE.^52^ Order of the WE and CE mixture depends on hydrocarbon chain length, branching, and saturation levels of both the CE and WE.^10^ The bulk meibum data from mixtures of CE and WE are relevant to meibum in the meibomian gland, and also to meibum on the tear film surface. Increased order and chain melting temperature of the bulk samples correlated with increases in the maximum surface pressure attained at minimal surface area and the transient dilatational modulus of the meibum layer at an air/water interface in vitro.^34^ Thus, changes in the spectroscopic packing parameters determined for bulk meibum translated to changes in the performance of the meibum layer at an air/water interface, and likely translate to changes in the surface film functionality of the TFLL. In future studies, it would be beneficial to separate meibum WE and CE and to measure WE and CE chain length, branching, and saturation using NMR spectroscopy, and then to compare the WE and CE hydrocarbon chain compositions to hydrocarbon chain order (conformation) using infrared spectroscopy, rheology,^53^ and Langmuir trough technology. A potential effect of aging of the WE and CE on hydrocarbon chain order could also be explored, as a study by Svitova and Lin^54^ demonstrated that aging of the lipid in model tear-lipid films slowed the rate of evaporation. One of the many advantages of using an NMR spectroscopic approach is that the sample is not destroyed upon compositional analysis as it is with mass spectrometry, and the sample can be used later for the quantification of structural order and elasticity using infrared spectroscopy and Langmuir trough technology, respectively. Not only the change in R_CE/WE_, but also changes in meibum saturation,^11^^,^^55^^,^^56^ hydrocarbon chain length^57^ and branching,^58^ protein levels,^59^ and (O-acyl)-ω-hydroxy fatty acid levels^57^^,^^60^ together or separately could contribute to dry eye, or at least be a marker for it.

In the current study, a 39% significant reduction in R_CE/WE_ was observed in the meibum from participants with MGD compared with no MGD. The analysis was performed in a masked manner, and the decrease in R_CE/WE_ observed in MGD was similar to the 40% reduction reported in a previous unmasked NMR study,^36^ and to the 28% reduction reported in an infrared study^37^; both of these previous studies used orders of magnitude more meibum than the current study. Although a small change in the level of CE was detected using spectrometry,^60^ changes in the R_CE/WE_ were not evident in other spectrometric studies^61^^,^^62^ possibly due to sample collection, donor demographic, and technical differences. Problems associated with one of the spectrometric studies^61^ have been addressed.^63^ The advantages of spectroscopic studies over spectrometry have also been addressed, as well as general issues related to spectrometric studies.^10^^,^^33^ It would be interesting to design a study using the same samples where different techniques were the only variable.

### NMR Evaluation Using a Limited Quantity of Meibum Sample

A recent study^35^ showed that the sum of the intensities of the 1 ppm and 0.63 ppm resonances from methyl moieties assigned to the cholesterol moiety in CE is six times more intense than when using the 4.6 ppm resonance from only one proton. We used this advantage in a post hoc analysis of the study spectra. As a result of the measurement improvements, all of the samples had measurable amounts of CE. In the post hoc analysis, we also more accurately integrated the 4.0 ppm triplet peak using a curve fitting algorithm. The results of the post hoc analysis were similar to those of the original analysis, showing a 39% decrease in R_CE/WE_ for meibum from participants with MGD compared with non-MGD participants (Table 3). In the post hoc analysis, R_CE/WE_ in meibum was numerically lower in the severe MGD cohort than in the mild-to-moderate MGD cohort and followed the trend non-MGD > mild-to-moderate MGD > severe MGD (Table 3).

Literature values of R_CE/WE_ in meibum from subjects without dry eye vary greatly from 0.3 to 0.94, with an outlier of 2.7 (Table 4^5^). An advantage of NMR spectroscopy is that the sample is not destroyed, so future studies can be designed to determine if the disparity in R_CE/WE_ across studies is due to methodology, innate variability, or sample demographics. The R_CE/WE_ in meibum samples from the non-MGD cohort measured in the post hoc analysis in this study, 0.38, was on the lower end of the published range of values, and almost identical to the R_CE/WE_ of 0.39 measured using FTIR spectroscopy with a different set of samples.^37^ An NMR study using a 500 mHz NMR reported a R_CE/WE_ of 0.57.^36^ Compared with 500 mHz NMR, the 700 mHz NMR used in the current study has better resolution because of the stronger magnetic field and is more sensitive because of the cold probe used.^35^ As sample sizes in the current study were very small, the evaluation would not have been feasible using a 500 mHz NMR instrument.

**Table 4.** Literature Values for the CE/WE Molar Ratio in Meibum From Donors Without Dry Eye

Citation	Molar Ratio of CE/WE	Number of Donors Sampled
Nicolaides et al.^18^	0.84	1 (76 pooled)
Tiffany et al.^78^	0.69	4
Brown et al.^23^	0.85	4
Chen et al.^33^	0.73	10
Lam et al.^61^	2.7 (Asian)	10
Borchman et al.^30^	0.54	72
Masoudi et al.^79^	0.72 (contact lens wearers)	27
Masoudi et al.^79^	0.30 (non-contact lens wearers)	30
Shrestha et al. ^36^	0.57	27
Butovich^24^	0.53	14
Chen et al.^20^	0.46	45
Hetman and Borchman^37^	0.39	47
Butovich et al.^63^^,^^*^	0.60	36
Butovich et al.^63^^,^^*^	0.45 (Asian)	38
Suzuki et al.^60^	0.94	24
Butovich and Suzuki^80^	0.52	50
Literature average (mean ± SEM for studies with n ≥ 10, excluding Lam et al.^61^)	0.56 ± 0.05	12 studies
Current study	0.38	22

CE, cholesteryl ester; SEM, standard error of the mean; WE, wax ester.

CE/WE ratios reported by weight were converted to CE/WE ratios by moles using a molecular weight (g/mole) of 734 for CE and 509 for WE.

* From % apparent abundance.

**Table 5.** Post Hoc Analysis of the CE/WE Molar Ratio Calculated Using the 1, 0.63, and 4.2 ppm Resonances in Samples With and Without a 4.6 ppm Resonance

	CE/WE Molar Ratio		
	Non-MGD	Mild-to-Moderate MGD	Severe MGD
No detectable 4.6 ppm resonance	0.21 ± 0.03 (9)	0.15 ± 0.06 (10)	0.18 ± 0.02 (19)
Detectable 4.6 ppm resonance	0.46 ± 0.06 (15)	0.34 ± 0.04 (11)	0.33 ± 0.05 (6)
*P*, no detectable vs detectable 4.6 ppm resonance	0.0094	0.0022	0.007

CE, cholesteryl ester; WE, wax ester.

Data are mean ± standard error of the mean. The number of samples is shown in parentheses.

Because resonances in the NMR spectra associated with the cholesterol moiety could result from either free cholesterol or CE in the meibum samples, it was not possible to use the NMR spectra to directly determine free cholesterol levels in the meibum. However, the finding that R_CE/WE_ calculated using the resonances at 1 and 0.63 ppm was no higher than R_CE/WE_ calculated using the CE resonance at 4.6 ppm suggests that free cholesterol was not measurable. Consistent with this finding, studies using high-pressure liquid chromatography/mass spectrometry have indicated that free cholesterol is only a minor component (0.5% to 1%) of human meibum.^24^^,^^64^ However, free cholesterol levels in human tears are higher,^24^^,^^62^ possibly because of cholesterol synthesis by the corneal epithelium.^65^ Locally made cholesterol, as well as CE from the meibomian glands in the tear film, may contribute to the high capacity of the corneal epithelium to heal from abrasions.^66^

### Parallels With Human Sebum

Among other factors, age influences meibum lipid homeostasis and the overall health of the meibomian glands, and the risk of developing MGD and evaporative DED increases with age.^67^^,^^68^ Histopathologic studies of human meibomian glands have shown that aging can be associated with meibomian gland atrophy, which results in MGD and DED.^67^^,^^69^^,^^70^ Interestingly, sebaceous glands, a type of holocrine gland found in the skin, like meibomian glands produce lipid-rich secretions (sebum) composed of various lipid species including WE, CE, squalene, and fatty acids.^71^^,^^72^ Studies have shown that age-related changes also result in sebaceous gland atrophy and skin diseases such as psoriasis and dermatitis.^73^^–^^75^ A recent study using comprehensive lipidomic analysis of meibum, sebum, and tears of a patient with abnormal meibomian and sebaceous gland secretions reported altered composition in various lipid classes in meibum, as well as sebum.^76^ Changes in the lipid composition of sebum, as well as meibum, could be important in ocular surface disease, because sebaceous gland secretions may also be incorporated into the tear film.^12^^,^^25^^,^^77^

Overall, the findings reported in this study suggest an important role of nonpolar lipids in MGD and pave the way for future studies to dissect the mechanism of altered CE/WE composition and factors affecting molar ratio variations of these lipids in the meibum of individuals with MGD/DED. Studies of meibum lipid hydrocarbon chain length, saturation, and branching, as well as quantification of the fatty acids in meibum using the NMR spectra from this study, are underway. The findings of these studies and the current spectroscopic analysis of meibum may potentially help identify new drug targets and therapeutic modalities for treatment of MGD and evaporative DED.

## Conclusion

This study used an NMR spectroscopic approach to evaluate meibum composition in participants with and without MGD. The results showed a significant decrease in R_CE/WE_ in individuals with MGD and suggest that R_CE/WE_ is associated with MGD disease severity. Changes in R_CE/WE_ are likely to influence hydrocarbon chain order and the rheology of the TFLL. It is reasonable to speculate that more ordered lipid (like butter) could inhibit the flow of meibum from the meibomian glands and contribute to the formation of a discontinuous patchy TFLL, which in turn results in deteriorated spreading and decreased surface elasticity. Furthermore, more ordered lipid could possibly result in an attenuated capability of restoring TFLL structure between blinks. These possibilities warrant further investigation. Finally, aside from the poor lipid quality in individuals who have MGD leading to tear film instability and evaporative tear loss, aldehydes in the abnormal meibum secretions may mix throughout the tears and potentially contribute to the chronic ocular surface inflammation that has been associated with DED.

## Supplementary Material

Supplement 1
