# Supplementary material for: Relationship Between Human Meibum Lipid Composition and the Severity of Meibomian Gland Dysfunction: A Spectroscopic Analysis
Source: Invest Ophthalmol Vis Sci. 2023 Jul 19;64(10):22. doi: 10.1167/iovs.64.10.22 (PMC10362926; doi:10.1167/iovs.64.10.22)
Supplement: Supplement 1 [file iovs-64-10-22_s001.pdf]

## Supplemental Methods

*Collection and Processing of Human Meibum*

The investigator collected meibum samples from the 6 central glands of the lower lid of each eye separately immediately after meibomian gland secretion quality was graded. An exclusive meibum collection kit (**Fig. S1**) was developed for use in the study.

*Preparation of Sebutape® for Use in Meibum Collection Kits*

To remove impurities from Sebutape® (Cuderm Corp., Dallas TX), the Sebutape® was removed from the cardboard backing, as supplied by the manufacturer, using sterile tweezers and a surgical needle (Fine point, Feilchenfeld Style Splinter Forceps, Electro-Optix, Inc). Surgical gloves were worn to prevent transfer of skin sebum to the Sebutape®. No more than 100 pieces of Sebutape® were placed into a clean 200 mL glass beaker that had been rinsed with 100 mL chloroform (HPLC grade, Sigma-Aldrich, St. Louis, MO) followed by 100 mL methanol (spectrophotometric grade, ACROS Organics, NJ). Chloroform (100 mL) was then added to the beaker containing the Sebutape® and the Sebutape®/chloroform was sonicated using a microprobe Sonifier® cell disrupter 185 (Branson, Ultrasonics Co., Danbury CT) for 15 seconds. The sonication was repeated twice more, 2 minutes between sonications to allow cooling. The chloroform was then removed and replaced with 100 mL of fresh chloroform, and the sonication steps described above were repeated 4 times in total, with fresh chloroform used at the beginning of each new cycle. The extracted Sebutape® was blotted on Kimwipe® tissues (Kimbery-Clark Corporation, Roswell GA) and dried in a lyophilizer set at 29 torr and a condenser temperature < −50°C (Freeze Dryer 3, Labconco, Kansas City, MO) for 1 hour to remove all solvent. Once dry, the Sebutape® was immediately used to prepare the meibum sample collection kits.

*Assembly of Meibum Collection Kits*

Assembly of the meibum collection device (**Supplemental Fig. S1**) was on clean weighing paper (Fisher Scientific, Pittsburgh PA, 6” X 6”, 09-898-12C). Surgical gloves were worn to avoid contamination due to finger sebum, and care was taken to never touch the Sebutape® to skin. The Sebutape® was positioned and handled with sterile tweezers (Fine point, Feilchenfeld Style Splinter Forceps, Electro-Optix, Inc.) Extracted, prepared Sebutape® was cut in half with scissors. The cardboard backing that had been removed was cut into 2 cm X 1 cm strips with one end rounded. The Sebutape® was placed onto weighing paper and folded (**Fig. S1A**), then the straight end of the cardboard was placed to cover half of the Sebutape®, and the tape was folded over the cardboard (**Fig. S1B**). Tweezers were used to secure the Sebutape® with the cardboard backing to an alligator clip (**Fig. S1C**). The cap liner of a scintillation vial cap was removed, and a nail was punched through the center of the cap liner, which was then glued to the cap with J-B Weld, ‘Waterweld’ glue (Model # 8277, J-B Weld, Inc, Sulphur Springs, TX) (**Fig. S1D**). The glue can withstand moisture and freezing. The alligator clip was then secured to the nail in the cap (**Fig. S1E**). The vial was filled with argon, and the cap assembly was screwed onto the vial (**Fig. S1F**).

*Sample Transportation from Clinical Sites and Short-term Sample Storage*

Meibum collection kits were shipped to the 3 clinical sites. After meibum collection, the samples for the study eyes were shipped for analysis to Department of Ophthalmology and Visual Sciences, University of Louisville, Louisville, KY on dry ice in sealed styrofoam containers. The samples were masked and did not contain information regarding the MGD status of the participant. A temperature recording device was included with the shipped samples to insure a constant low temperature was achieved. Care was taken to log the arrival of the samples in the department delivery log. No sample vials were cracked or damaged during shipment. Samples were stored in a locked, dedicated −20˚C freezer.

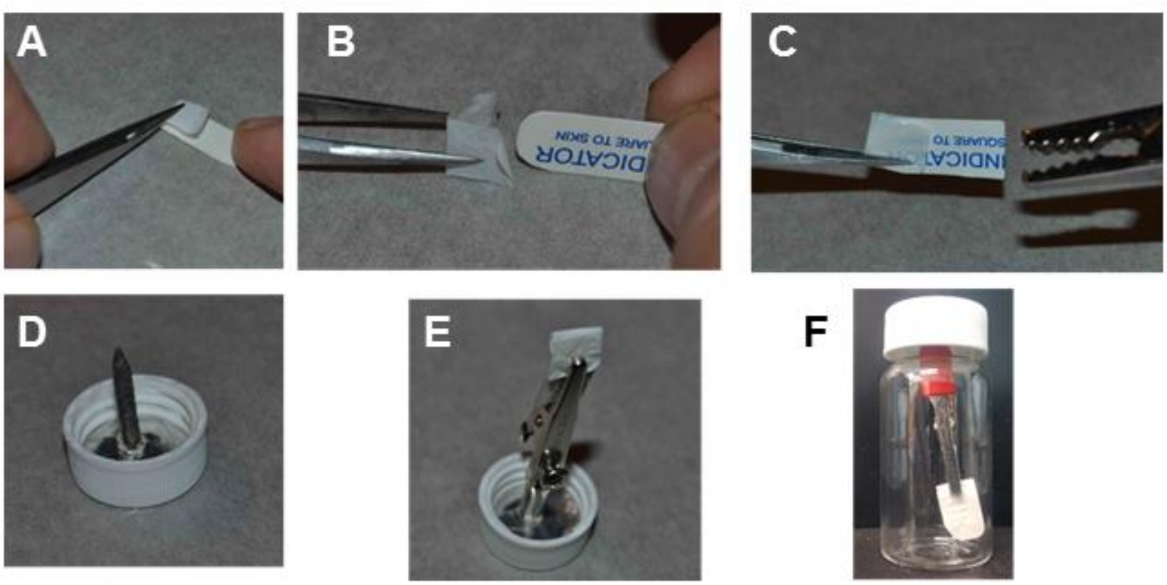


**Supplemental Figure S1. Assembly of the Sebutape® (Cuderm Corporation, Dallas, TX) meibum collection kit.** (**A**) Sebutape® folded. (**B**) Sebutape® positioned onto cardboard backing. (**C**) Sebutape® fastened to the cardboard backing with an alligator clip. (**D**, **E**) Nail to hold the alligator clip assembly glued to a scintillation vial cap. (**F**) Final Sebutape® meibum collection kit assembly: the scintillation vial cap with alligator clip assembly is secured to a scintillation vial with the folded edge of the Sebutape® suspended approximately 1 cm above the bottom of the vial, which is filled with argon gas to protect the meibum from oxidation. For use, the investigator touched the Sebutape® to the eyelid where the meibum was expressed and placed the Sebutape® and meibum sample back into the sealed vial. Meibum lipid was later extracted from the Sebutape®, and the meibum lipid composition was quantified using NMR spectroscopy.
